# Elucidation of Selective Apoptosis of Breast Cancer Cells Induced by *Panax ginseng*–Derived Exosomes via Regulation of Bax/Bcl‐2 Ratio and AMPK‐*α* Signaling

**DOI:** 10.1155/ijbc/3346672

**Published:** 2026-10-22

**Authors:** Min Ho Han, Youn Seon Hwang, Jong Hyun Oh, Do Hyeon Jin, Ju Hwan Lee, Jin Woo Kim

**Affiliations:** ^1^ Department of Food Technology & Nutrition Sunmoon University Asan-si Chungcheongnam-do Republic of Korea sunmoon.ac.kr

**Keywords:** anticancer, apoptosis, breast cancer, exosome, *Panax ginseng*

## Abstract

This study evaluated the anticancer potential of *Panax ginseng*–derived exosomes (PGEs) against MDA‐MB‐231 breast cancer cells, focusing on AMP‐activated protein kinase (AMPK)–mediated apoptosis and tumor cell selectivity. PGE was isolated using size‐exclusion chromatography and exhibited a mean particle size of 163.4 nm with a concentration of 1.2 × 10^10^ particles/mL. The exosomal identity was verified by elevated expression levels of CD9 (91.4%), CD63 (96.8%), and TET‐8 (127.2%). Treatment with PGE caused a dose‐dependent decline in the viability of MDA‐MB‐231 cells (87.2% at 0.38 × 10^10^ particles/mL), while normal human mammary epithelial cells (HMECs) maintained 90.1% viability even at 3.0 × 10^10^ particles/mL, indicating selective induction of apoptosis. In scratch assays, PGE suppressed MDA‐MB‐231 migration by up to 29.8% but enhanced HMEC proliferation and migration by 88.5%, demonstrating selective anti‐invasive effects. Molecular analysis showed increased expression of AMPK‐*α*, Bax, caspase‐3, and caspase‐9, coupled with downregulation of Bcl‐2 and vascular endothelial growth factor, thereby validating mitochondrial apoptosis as the underlying mechanism. Flow cytometry further revealed a dose‐dependent increase in early and late apoptosis in MDA‐MB‐231 cells (total apoptosis: 17.7%–32.9%), while HMEC displayed only minimal apoptotic response (< 7.6%). Immunofluorescence analysis also indicated chromatin condensation, ZO‐1 disruption, and F‐actin reorganization in MDA‐MB‐231, whereas HMEC preserved structural integrity with enhanced ZO‐1 and F‐actin intensity. LC‐MS/MS profiling identified ginsenosides Rf, Rh1, and Rb1 as principal bioactive constituents of PGE, implicating them in AMPK‐dependent apoptosis. Taken together, these results demonstrated that PGE selectively triggers apoptosis in breast cancer cells while maintaining normal cell viability, highlighting its potential as a natural exosome‐based anticancer therapeutic with strong biocompatibility and translational potential.

## 1. Introduction

Cancer is defined by uncontrolled cellular proliferation and metastatic spread and remains one of the leading global causes of morbidity and mortality. According to the American Cancer Society Global Cancer Statistics 2024, cancer caused approximately 9.7 million deaths, and this number is projected to rise to 35 million by 2050 [1]. Among malignancies, breast cancer is the most common cancer in women and the second leading cause of cancer‐related mortality among women. This mortality rate has increased steadily by 1.4% per year, with 517,900 deaths reported in 2022, underscoring its clinical importance [2]. Originating within mammary tissue, breast cancer shows pronounced heterogeneity and a complex tumor microenvironment, both contributing to high recurrence rates [3]. Early detection of atypical hyperplasia remains difficult, limiting opportunities for timely diagnosis. Clinically, breast cancer is classified by the expression of estrogen receptor (ER), progesterone receptor (PR), and human epidermal growth factor receptor 2 (HER2), which serve as essential molecular markers for prognosis and therapeutic decisions [4].

In cancer cells, apoptosis is regulated by multiple intracellular signaling cascades, including the mitochondrial, AMP‐activated protein kinase (AMPK), and mitogen‐activated protein kinase (MAPK) pathways. Of these, AMPK is activated under reduced intracellular ATP levels and serves as a central metabolic regulator that preserves energy balance, suppresses growth, and promotes autophagy [5]. Once activated, AMPK enhances the proapoptotic activity of B‐cell lymphoma 2‐associated X (Bax), which translocates into the mitochondrial outer membrane, increases permeability, and facilitates the release of cytochrome c (Cyt c) into the cytoplasm. Cyt c then binds apoptotic protease‐activating factor 1 to form the apoptosome, which, in turn, activates cysteine‐dependent aspartate‐directed protease (caspase) 9. This cascade culminates in downstream caspase‐3 activation, leading to nuclear condensation, DNA fragmentation, and membrane blebbing, all classical hallmarks of apoptosis [6]. In contrast, B‐cell lymphoma 2 (Bcl‐2) functions as an antiapoptotic protein that antagonizes Bax, prevents mitochondrial binding, and blocks Cyt c release, favoring cell survival. Thus, modulation of AMPK signaling represents a strong therapeutic strategy to reduce Bcl‐2 expression while promoting Bax activation, thereby inducing intrinsic mitochondrial apoptosis in cancer cells.

Currently used chemotherapeutics for breast cancer include doxorubicin, cisplatin, and paclitaxel, each of which exerts cytotoxic effects by disrupting DNA replication, forming covalent adducts, or stabilizing microtubules. However, these drugs are associated with severe adverse effects such as myelosuppression, peripheral neuropathy, and gastrointestinal toxicity, highlighting the need for safer yet effective alternatives [7, 8]. Natural bioactive compounds capable of selectively inducing apoptosis and targeting the tumor microenvironment have therefore gained increasing attention. *Panax ginseng* contains ginsenosides, a class of triterpenoid saponins with immunomodulatory activities [9]. Ginsenosides have been shown to activate AMPK signaling, thereby inducing autophagy‐dependent apoptosis and suppressing tumor growth [10]. Nonetheless, phytochemicals often exhibit adverse effects such as enzymatic degradation, poor bioavailability, and limited tissue‐specific delivery during in vivo metabolism, which restrict their therapeutic effectiveness [11].

Exosomes are nanoscale extracellular vesicles (50–200 nm) derived from multivesicular bodies that encapsulate bioactive molecules, including DNA, mRNA, proteins, and lipids. They play crucial roles in intercellular communication and maintenance of tissue homeostasis [12]. Exosomes are enriched in tetraspanin proteins such as Cluster of Differentiation (CD) 9, CD63, and CD81, which mediate cell–cell interactions, direct cargo delivery, and regulate gene expression, ultimately affecting immune responses and differentiation [13]. Recent findings further suggest that exosomal membrane proteins enhance biocompatibility and coordinate signal transduction in the tumor microenvironment, thereby influencing cancer progression and metastasis [14]. Although *Panax ginseng*–derived exosomes (PGEs) have been reported to exert antioxidant effects, promote wound healing, and reduce skin aging, their anticancer efficacy in breast cancer remains poorly defined. In particular, the role of PGE in modulating AMPK‐*α* signaling and inducing apoptosis in breast cancer cells has not yet been clarified. Accordingly, this study investigates the proapoptotic effects of PGE in breast cancer via the regulation of the AMPK‐*α* axis, aiming to provide a mechanistic basis for their development as natural exosome‐based anticancer therapeutics.

## 2. Materials and Methods

### 2.1. Callus Culture and Exosome Isolation

For this study, we obtained 4‐year‐old *Panax ginseng* plants cultivated in Eumseong, Chungbuk, Korea; their roots and stems were separated. The roots were rinsed with distilled water, surface‐sterilized with 70.0% (*v*/*v*) ethanol, and immersed in 2.0% sodium hypochlorite solution for 10 min. Then, they were washed three times with sterile distilled water. To induce ginseng‐derived callus, root segments (3.0–4.0 cm) were cultured on Murashige and Skoog (MS) medium (Duchefa Biochemie, Haarlem, Netherlands) containing 1.0 mg/L 2,4‐dichlorophenoxyacetic acid (2,4‐D, Duchefa Biochemie), 3.0% (*w*/*v*) sucrose (Duchefa Biochemie), and 0.3% (*w*/*v*) Gelrite (Duchefa Biochemie). Cultures were maintained in the dark at 23.0°C with 60%–70% relative humidity. They were then subcultured every 2 weeks using MS medium filtered through a 1.2 *μ*m filter (Cytiva, Little Chalfont, United Kingdom). We first isolated exosomes from the conditioned callus culture medium using size‐exclusion chromatography (SEC) with an ÄKTA fast protein liquid chromatography system (Cytiva). A HiPrep 26/10 Desalting column (Cytiva) was used as the stationary phase, and 20.0 mM sodium phosphate buffer (pH 6.0) served as the mobile phase. The culture medium was applied to the column, fractions were collected at a flow rate of 1.0 mL/min with 1.0 mL per fraction, and the samples were further filtered through a 0.44 *μ*m membrane filter. Purified fractions were then stored at −20.0°C to preserve exosome integrity.

### 2.2. Characterization of Exosomes

To characterize isolated PGE, nanoparticle tracking analysis (NTA) was performed to monitor the Brownian motion of individual nanoparticles via laser scattering. Analyses were conducted with a ZetaView platform (PMX 130, Particle Metrix GmbH, Meerbusch, Germany) equipped with a 488 nm laser. Before imaging, PGE samples were diluted 1:100 (*v*/*v*) in ultrapure distilled water prefiltered through a 0.1 *μ*m membrane filter (Hyundai Micro Co., Ltd.) to minimize particle overlap and multiple scattering. Measurements were conducted in triplicate under the following conditions: electrolyte, distilled water; temperature, 23.1°C (sensed); pH, 7.0 (entered); and conductivity, 15.0 *μ*S/cm (sensed). For all trials, the instrument parameters were set to the following values: scatter detection mode, sensitivity 80, shutter speed 100, maximum area 1000, minimum area 10, minimum brightness 30, and bin size 5 nm/class. Particle size distribution was then calculated across 11 diameter‐based intervals using one measurement cycle covering 11 positions, with an average of 76 particles per frame and 631 particles successfully tracked. Hydrodynamic diameter was calculated using the Stokes–Einstein equation, and particle concentrations were corrected for dilution. To confirm plant‐derived exosomal identity, PGE was recovered with ExoQuick‐TC, followed by protein extraction and western blot analysis of exosomal biomarkers, including CD9, CD63, and Tetraspanin‐8 (TET‐8).

### 2.3. Cell Line Culture

Human mammary epithelial cells (HMECs) and the human breast adenocarcinoma cell line MDA‐MB‐231 were obtained from the American Type Culture Collection (ATCC, Manassas, Virginia, United States) and the Korean Cell Line Bank (Seoul, Korea), respectively. Cells were cultured in Dulbecco’s Modified Eagle Medium (DMEM): Nutrient Mixture F12 supplemented with 10.0% (*v*/*v*) fetal bovine serum (FBS) and 1.0% (*v*/*v*) penicillin–streptomycin solution. Both cell lines were seeded at a density of 2.5 × 10^4^ cells/mL in culture flasks and maintained at 37.0°C in a humidified incubator (MCO‐5AC, Sanyo, Osaka, Japan) under 5.0% CO_2_. All culture media and reagents used for cell culture and subsequent experiments were purchased from Thermo Fisher Scientific (Waltham, Massachusetts, United States) and were of analytical grade or higher.

### 2.4. Cell Viability Assay

Cell viability was assessed based on the reduction of 3‐(4,5‐dimethylthiazol‐2‐yl)‐2,5‐diphenyltetrazolium bromide (MTT) [15]. HMECs and MDA‐MB‐231 cells were seeded into 96‐well plates at a density of 1.0 × 10^5^ cells/well and incubated for 24 h. Subsequently, cells were treated with PGE (0.0–6.0 × 10^10^ particles/mL) and further incubated for an additional 24 h. After treatment, 0.6 *μ*M MTT solution was added to each well and incubated for 4 h. The resulting formazan crystals were solubilized using dimethyl sulfoxide (DMSO), and absorbance was measured at 450 nm using a microplate reader (AMR‐100, Allsheng, Hangzhou, Zhejiang, China). Cell viability was calculated as a percentage according to Equation (1). Dose–response curves were further used to estimate IC20–IC50 values by nonlinear regression.

(1) Cell viability %= absorbance of PGE−treated groupabsorbance of non−treated control group ×100.

### 2.5. Cell Migration Assay

Cell migration was evaluated to assess the inhibitory effect of PGE on cancer cell metastasis using a scratch wound healing assay. HMECs and MDA‐MB‐231 cells were seeded into 24‐well plates at a density of 1.0 × 10^5^ cells/mL and incubated for 24 h. A linear scratch of approximately 1.0 mm in width was created in the cell monolayer using a sterile pipette tip, and detached cells were removed by washing twice with phosphate‐buffered saline (PBS). Cells were then treated with PGE at concentrations ranging from 0.0 to 3.8 × 10^9^ particles/mL. Cell migration was monitored at 12 h intervals (0, 12, and 24) using an optical microscope (CX43, Olympus, Tokyo, Japan). The wound area at each time point was quantified using ImageJ software (National Institutes of Health, Bethesda, Maryland, United States). Cell migration was calculated as a percentage according to Equation (2).

(2) Cell migration %= 1−wound area of PGE−treated group initial wound area of non−treated group 0 h ×100.

### 2.6. Gene Expression Analysis

To evaluate whether apoptosis is a key mechanism regulating PGE activity, HMECs and MDA‐MB‐231 cells were treated with PGE (0.0–3.8 × 10^9^ particles/mL) and incubated for 24 h. Total RNA was then extracted using an Accuprep Universal RNA Extraction Kit (Bioneer, Daejeon, Korea). Complementary DNA (cDNA) was synthesized from 1.0 *μ*g of RNA using the cDNA Synthesis Platinum Master Mix (GenDEPOT, Barker, United States). RNA was pretreated with diethyl pyrocarbonate (DEPC; Bioneer) to prevent degradation. Semiquantitative reverse transcription‐polymerase chain reaction (qRT‐PCR) was conducted using primers specific for apoptosis‐related genes, including *Bax*, *Bcl-2*, *caspase-3*, *caspase-9*, AMPK‐*α*, and vascular endothelial growth factor (VEGF) (Table 1). Products were then loaded onto 1.5% (*w*/*v*) agarose gels containing GelRed nucleic acid stain (Komabiotech, Seoul, Korea) and separated by electrophoresis. Band intensities were finally visualized with a Davinch gel imager (CG‐550, Davinch‐K, Seoul, Korea) and quantified using CLIQS 1D software (Ver. 1.6.454, TotalLab, Newcastle upon Tyne, United Kingdom).

**Table 1:** Primer sequences for semiquantitative reverse transcription‐polymerase chain reaction analysis of key anticancer genes in HMEC and MDA‐MB‐231.

Genes	Forward (5 ^′^‐3 ^′^)	Reverse (5 ^′^‐3 ^′^)
*Bax*	TGTTTTCTGACGGCAACTTCA	CAGTTCCGGCACCTTGGT
*Bcl-2*	CATGTGTGTGGAGAGCGTCAA	GACAGCCAGGAGAAATCAAACAG
*Caspase-3*	TACCAGTGGAGGCCGACTTC	CAAAGCGACTGGATGAACCA
*Caspase-9*	AACAGGCAAGCAGCAAAGTTG	CTGCTCGACATCACCAAATCC
AMPK‐*α*	GACACCAGTTTTGCCTCCAGTA	TCCAGAGGCGGAAGTTCTGT
*VEGF*	GCTGTCTTGGGTGCATTGG	GCAGCCTGGGACCACTTG
*GADPH*	GATGGGCATGAAGCATGAGA	TGGCATGGACTGTGGTCATT

Abbreviations: *AMPK-α*, AMP‐activated protein kinase‐*α*; *Bax*, Bcl‐2 associated x; *Bcl-2*, B‐cell lymphoma‐2; *caspase-3*, cysteine‐dependent aspartate‐directed protease 3; *caspase-9*, cysteine‐dependent aspartate‐directed protease 9; *GADPH*, glyceraldehyde‐3‐phosphate dehydrogenase; *VEGF*, vascular endothelial growth factor.

### 2.7. Protein Expression Analysis

To assess whether transcriptional alterations resulted in altered protein levels, apoptosis‐related protein expression was analyzed in MDA‐MB‐231 cells and HMECs treated with PGE (0.0–3.8 × 10^9^ particles/mL) and incubated for an additional 24 h under identical conditions. Subsequently, total protein was extracted using a radioimmunoprecipitation assay buffer following supplementation with a protease inhibitor cocktail. Concentrations were determined using the Bradford assay. Equal protein amounts (30.0 *μ*g per sample) were resolved on 12.0% (*w*/*v*) sodium dodecyl sulfide–polyacrylamide gels and transferred onto polyvinylidene difluoride membranes. To prevent nonspecific binding, membranes were blocked with 5.0% (*w*/*v*) skim milk for 1 h at room temperature and then washed with PBS supplemented with 0.1% (*v*/*v*) Tween‐20 (PBS‐T). Primary antibodies against apoptosis‐related proteins and exosomal biomarkers, along with secondary antibodies, were diluted in 5.0% (*w*/*v*) skim milk (1:100–1:2000, *v*/*v*) and applied sequentially. Protein expression was visualized with Enhanced Chemiluminescence (Kindle Bio, Greenwich, Connecticut, United States) and quantified using a KwikQuant Pro Multi‐Image system (D1010, Kindle Bio) and CLIQS 1D software (TotalLab). Finally, band intensities were normalized to *β*‐actin as a loading control.

### 2.8. Apoptosis Analysis by Flow Cytometry

Next, we performed flow cytometry with a Dead Cell Apoptosis Kit, which contains Annexin V and propidium iodide (PI) (Thermo Fisher Scientific). MDA‐MB‐231 cells and HMECs were seeded into six‐well plates at a density of 1.0 × 10^5^ cells/well and incubated for 24 h, followed by treatment with PGE (0.0–3.8 × 10^9^ particles/mL) for another 24 h. Cells were harvested, washed twice with cold PBS, and resuspended in binding buffer. Annexin V–fluorescein isothiocyanate (FITC) and PI were added, and samples were incubated for 15 min at room temperature in the dark. Additional binding buffer was then added, and fluorescence signals were acquired using a CytoFLEX V0‐B4‐R0 flow cytometer (Beckman Coulter, Brea, California, United States). Data were analyzed with CytExpert software (Ver. 2.5.0.77, Beckman Coulter) using forward scatter/side scatter (FSC/SSC) gating to exclude debris and doublets. Cells were classified into four populations: viable (Annexin V^−^/PI^−^), early apoptotic (Annexin V^+^/PI^−^), late apoptotic (Annexin V^+^/PI^+^), and necrotic (Annexin V^−^/PI^+^).

### 2.9. Immunofluorescence Analysis

Overall, we used the results of our immunofluorescence analysis project to examine structural changes in MDA‐MB‐231 and HMEC cells related to Bax/Bcl‐2 and AMPK‐*α* pathway activation. Accordingly, cells were first seeded in 24‐well plates at 2.0 × 10^5^ cells/well and incubated for 24 h, followed by PGE treatment and an additional 24 h incubation. After PBS washing, cells were fixed with 4.0% paraformaldehyde for 30 min and permeabilized with 0.1% Triton X‐100 for 10 min. To block nonspecific binding, cells were incubated with 0.2% (*w*/*v*) bovine serum albumin (BSA) for 30 min. Primary antibody against zonula occludens‐1 (ZO‐1; Thermo Fisher) was applied overnight at 4.0°C. In parallel, the actin cytoskeleton was stained with phalloidin‐iFluor 488 (F‐actin, Thermo Fisher). After PBS washes, Alexa Fluor 594–conjugated secondary antibodies (Thermo Fisher) were added for 1 h at 4.0°C in the dark, followed by nuclear counterstaining with 4 ^′^,6‐diamidino‐2‐phenylindole (DAPI; Thermo Fisher) for 10 min. Fluorescence images were obtained using an inverted microscope (CKX53, Olympus, Shinjuku, Tokyo, Japan) with consistent exposure and gain. Protein localization and fluorescence intensity were quantified with ImageJ software (NIH, Bethesda, Maryland, United States). Fluorescence intensity was calculated as integrated density divided by the number of DAPI‐positive nuclei per field after background subtraction.

### 2.10. Ginsenoside Analysis by LC‐MS/MS

For qualitative and quantitative ginsenoside profiling, PGE samples were lyophilized with a freeze dryer (FD‐8, Daihan Scientific, Seoul, Korea) at −80.0°C and 3.0 Torr for 72 h. The resulting powder was dissolved in 70.0% (*v*/*v*) methanol and filtered through a 0.22 *μ*m membrane (Hyundai Micro Co., Ltd., Seoul, Korea) before analysis. Profiling was conducted with a Vanquish Flex UHPLC system (Thermo Fisher Scientific) using a ROC C18 column (3.0 × 150 mm, Restek Ltd., Pennsylvania, United States) coupled to a TSQ Altis Plus mass spectrometer (Thermo Fisher Scientific). Four ginsenosides (Rf, Rb1, Rb2, and Rh1) were analyzed simultaneously. Identification was based on retention time, mass‐to‐charge ratio (*m*/*z*), fragment ions, and peak area. Quantification used calibration curves constructed in multiple reaction monitoring (MRM) mode. Analyses were performed under electrospray ionization (ESI) in both positive and negative ion modes with optimized collision energies. The mobile phases were HPLC‐grade acetonitrile (JT Baker, Phillipsburg, New Jersey, United States), ultrapure water (Milli‐Q IQ7000, Merck, Darmstadt, Germany), and formic acid (Alfa Aesar, Haverhill, United States). Final ginsenoside concentrations were expressed as milligrams per kilogram of sample.

### 2.11. Statistical Analysis

All experiments were performed in triplicate, and data are expressed as the mean ± standard deviation. Statistical analyses were conducted using GraphPad Prism software (Version 10.4.1; GraphPad Software, San Diego, California, United States). Differences between two groups were analyzed using an unpaired Student’s *t*‐test, while comparisons among multiple groups were evaluated using one‐way analysis of variance (ANOVA) followed by Tukey’s post hoc test. Statistical significance was considered at  ^∗^ *p* < 0.05,  ^∗∗^ *p* < 0.01, and  ^∗∗∗^ *p* < 0.001.

## 3. Results and Discussion

### 3.1. Exosome Characterization

Exosomes are nanoscale vesicles released from cells that mirror the physiological and pathological status of their origin. This feature makes them promising diagnostic biomarkers for specific diseases. Due to their intrinsic mobility and lipid bilayer, which protects enclosed cargo, exosomes have also drawn attention as next‐generation drug delivery carriers. For their application as biomarkers or therapeutics, it is essential to develop methods capable of reliably producing high‐purity exosomes at scale. Accordingly, PGEs were isolated by SEC, and their physicochemical features were confirmed through NTA and biomarker profiling.

The mean diameter of exosomes isolated by SEC was 163.4 nm, within the typical range of 50–200 nm (Figure 1A). ZetaView analysis confirmed the particle distribution, indicating successful vesicle isolation. Expression of canonical markers CD9 and CD63 increased by 91.4% and 96.8% relative to those values observed in the culture medium (Figure 1C,D). Taken together, these findings confirm exosomal identity (Figure 1C,D, *p* < 0.001). In addition, expression of TET‐8, a plant‐specific exosomal marker involved in biogenesis and signaling, rose by 127.2% in PGE compared with culture medium, supporting their plant origin (Figure 1E). The exosome concentration was 1.2 × 10^12^ particles/mL, approximately 125.1–701.8‐fold higher than that of *Solanum nigrum* L. exosomes (8.1 × 10^9^) isolated by ultrafiltration, *Vaccaria hispanica* exosomes (9.59 × 10^9^) obtained by polyethylene glycol (PEG) precipitation, and *Nicotiana tabacum* exosomes (1.71 × 10^9^) recovered by ultracentrifugation and PEG [16–18]. These data show that SEC enables exosome isolation at a higher yield than conventional methods. Importantly, SEC also removed nonexosomal contaminants such as apoptotic debris, amino acid aggregates, and lipid particles while preserving vesicle structure, allowing recovery of samples with both high purity and yield.

**Figure 1:**
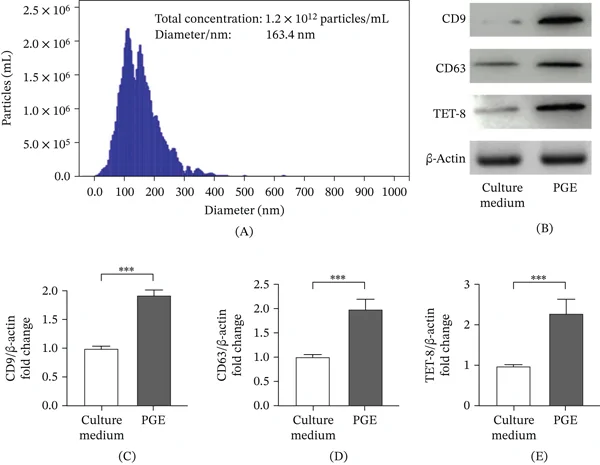
Characterization of *Panax ginseng*–derived exosomes (PGEs). (A) Nanoparticle tracking analysis, particle size distribution and concentration. (B) Western blot analysis of exosomal markers. Quantitative analysis of (C) CD9, (D) CD63, and (E) TET‐8 expression normalized to *β*‐actin in PGE. Statistical significance was determined by unpaired Student’s *t*‐test.

Traditional isolation approaches include ultracentrifugation, density gradient centrifugation, PEG precipitation, and immunoaffinity chromatography. In contrast, SEC separates particles by size through porous chromatographic columns, reducing mechanical damage and producing exosomes of a defined range and higher purity [19]. Thus, SEC is a scalable and robust method that provides yield and purity superior to conventional techniques. It also offers potential for lowering production costs and improving efficiency in biomaterial and pharmaceutical development [20]. SEC‐based isolation is therefore a key enabling technology that can accelerate translation in immunotherapy, drug delivery, and diagnostics. Within this framework, we investigated the effects of PGE on apoptosis and prodeath signaling in breast cancer cells to clarify their role as anticancer bioactive agents.

### 3.2. Cell Viability Analysis

Breast cancer cells are central drivers of tumor growth and metastasis in vivo, where early tumor microenvironments activate survival and proliferation pathways. In contrast, the AMPK‐*α* pathway regulates energy balance and, under metabolic stress, suppresses growth and induces autophagy, thereby restraining proliferation [21]. To determine whether PGE selectively promoted cancer cell death, we compared viability changes in MDA‐MB‐231 cells and HMEC after treatment.

Overall, we observed that PGE treatment reduced MDA‐MB‐231 viability in a dose‐dependent manner (Figure 2A). At 0.38 × 10^10^ particles/mL, viability was 86.6%, while at 0.75 × 10^10^ and 1.5 × 10^10^ particles/mL, it dropped to 84.0% and 83.1%, respectively (Figure 2A). In contrast, HMEC exposed to 3.0 × 10^10^ particles/mL retained 90.1% viability with no significant decrease (*p* > 0.05), indicating that PGE did not impair normal cells at this level. At 6.0 × 10^10^ particles/mL, HMEC viability fell significantly, likely reflecting cytotoxicity from high concentrations of bioactive compounds (Figure 2A). To avoid confounding by toxicity in normal cells, subsequent assays used 0.38 × 10^10^ particles/mL. This distinction emphasizes that, unlike many phytochemical extracts, PGE does not appear to harm normal cells.

**Figure 2:**
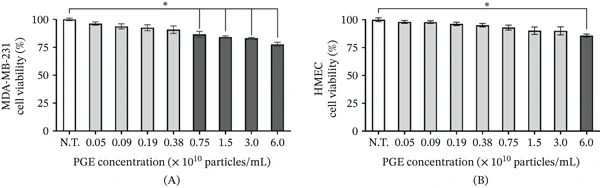
Selective effects of *Panax ginseng*–derived exosome (PGE) on cell viability measured by MTT assay in (A) MDA‐MB‐231 and (B) HMEC. Values normalized to the nontreated group.

Previous studies also showed that PGE suppressed proliferation and induced apoptosis in MCF‐7 breast cancer cells, supporting significant anticancer properties [22]. A comparable inhibitory effect was seen here in MDA‐MB‐231 cells, further confirming suppression of cancer cell viability. Overall, these findings demonstrate that, relative to other plant‐derived exosomes, PGE maintains normal cell viability and offers strong biocompatibility alongside therapeutic promise.

Based on nonlinear regression analysis of dose–response curves using a four‐parameter logistic model, the estimated IC20 and IC50 values for MDA‐MB‐231 cells were approximately 7.6 × 10^9^ and 49.9 × 10^9^ particles/mL, respectively, whereas HMEC exhibited markedly higher tolerance with estimated IC20 and IC50 values of ~26.8 × 10^9^ and ~611.0 × 10^9^ particles/mL [23]. Accordingly, all mechanistic experiments were conducted well below the IC20 threshold, minimizing nonspecific cytotoxicity while preserving pathway‐specific interpretability. Although this study focused on a single triple‐negative breast cancer model, MDA‐MB‐231 was selected for its well‐characterized dysregulation of metabolic and apoptotic signaling pathways [24]. Future studies will extend validation to additional breast cancer subtypes and in vivo models to assess generalizability.

### 3.3. Cell Migration

In the early stages of breast cancer progression, tumor cells secrete matrix metalloproteinases (MMPs) that degrade the extracellular matrix (ECM), initiating invasion [25]. Through the epithelial–mesenchymal transition, cancer cells acquire a mesenchymal phenotype with increased motility and invasiveness relative to normal epithelial cells, thereby accelerating tumor development [26]. Within the tumor microenvironment, proliferation and survival are regulated, and these processes connect to apoptosis through caspase‐3 and caspase‐9 activation, which are, in turn, controlled by Bcl‐2 and Bax.

In migration assays, both cell types under nontreated (N.T.) conditions showed gradual wound gap reduction over time. Changes in the wound area at 24 h were used to compare motility and invasive capacity between cancer and normal cells (Figure 3A,C). In MDA‐MB‐231 cells, treatment with PGE at 0.9 × 10^9^ particles/mL reduced wound closure by 21.1% at 12 h and 29.8% at 24 h relative to N.T., confirming inhibition of migration (Figure 3B). After 24 h, treatment with 1.9 × 10^9^ and 3.8 × 10^9^ particles/mL reduced wound closure to 37.1% and 55.4%, respectively, in a concentration‐dependent manner. These results suggest that PGE suppresses motility by interfering with proliferation‐ and adhesion‐related pathways.

**Figure 3:**
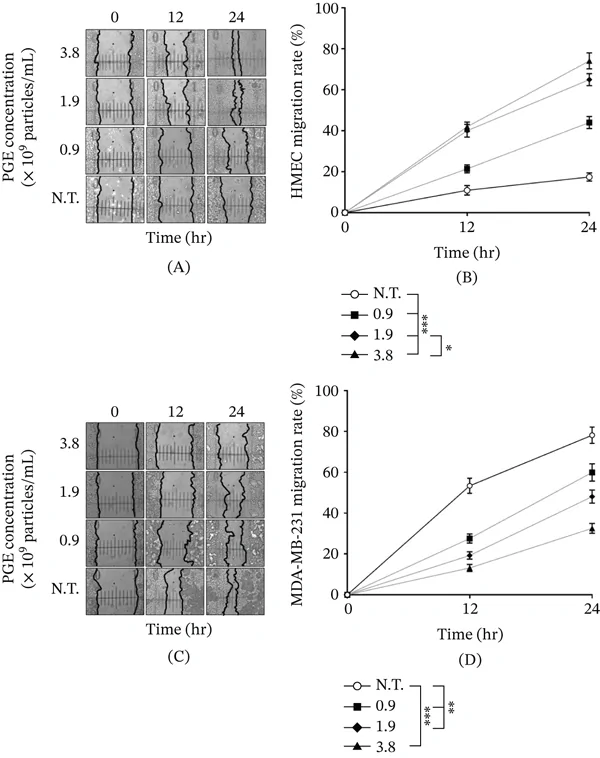
Scratch assay–based evaluation of cell migration in HMEC and MDA‐MB‐231 cells following treatment with Panax ginseng‐derived exosomes (PGEs). Representative scratch assay images of (A) HMEC and (C) MDA‐MB‐231 cells at 0, 12, and 24 h after treatment with PGEs at concentrations of 0.9, 1.9, and 3.8 × 10⁸ particles/mL. Quantification of the migration rates (%) of (B) HMEC and (D) MDA‐MB‐231 cells.

To test selectivity, migration was also examined in normal cells. In HMEC, treatment with 0.9 × 10^9^ particles/mL PGE increased wound closure to 77.2% at 12 h and 88.5% at 24 h (Figure 3D). This shows enhanced proliferation while cancer cell migration was inhibited. At 1.9 × 10^9^ and 3.8 × 10^9^ particles/mL, wound closure reached 75.1% and 85.6%, respectively, demonstrating that PGE supports survival and proliferation of normal cells. Collectively, these findings emphasize that PGE can act as a selective anticancer agent by suppressing migration in cancer cells and preserving normal cell activity. Next, to confirm this mechanism, expression of apoptosis‐related mediators was next examined.

### 3.4. Gene Expression

Apoptosis is a fundamental regulator of cancer cell growth and proliferation, governed by multiple signaling pathways. Bcl‐2 and Bax are key regulators of the mitochondrial apoptosis pathway by controlling mitochondrial outer membrane permeabilization. Cyt c release then promotes apoptosome formation and activation of the initiator caspase‐9, which in turn activates the executioner caspase‐3 to drive the apoptotic cascade [27]. In addition, AMPK‐*α* maintains energy balance and under metabolic stress promotes apoptosis. This study examined whether PGE induced apoptosis in cancer cells through modulation of the Bax/Bcl‐2 ratio, caspase activity, and AMPK‐*α* pathway regulation.

In MDA‐MB‐231 cells (Figure 4A–F), PGE treatment increased Bax expression by 6.6%–28.9% relative to N.T., while Bcl‐2 expression decreased by 12.1%–26.3%, elevating the Bax/Bcl‐2 ratio by 1.2–1.8‐fold (Figure 4A,B). These findings suggest that PGE enhanced mitochondrial permeability and activated intrinsic apoptosis. Caspase‐3 and caspase‐9 expression rose by 1.7%–25.3% and 1.0%–11.8%, respectively, confirming progression to the execution phase (Figure 4C,D). AMPK‐*α* expression increased by 3.3%–16.1%, suggesting that PGE triggered metabolic stress responses coupling energy homeostasis with apoptotic signaling (Figure 4E). VEGF expression declined by 6.9%–30.1% in a dose‐dependent manner, indicating that PGE simultaneously induced apoptosis and inhibited angiogenesis (Figure 4F).

**Figure 4:**
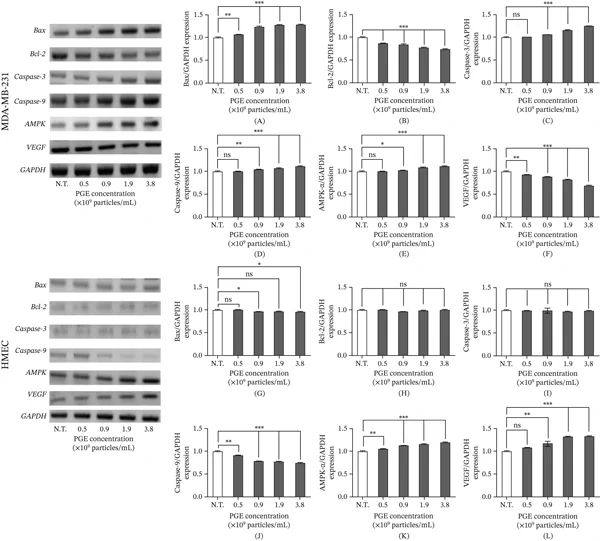
Evaluation of apoptosis‐related gene expression in (A–F) MDA‐MB‐231 and (G–L) HMEC cell following treatment with *Panax ginseng*–derived exosome (PGE). Expression levels of (A, G) *Bax*, (B, H) *Bcl-2*, (C, I) *caspase-3*, (D, J) *caspase-9*, (E, K) *AMPK-α*, and (F, L) *VEGF* were analyzed to assess mitochondrial apoptosis activation.

In contrast, HMEC treated with PGE (≤3.8 × 10^9^ particles/mL) showed no significant apoptotic changes (Figure 4G–4L). Bax expression decreased by 4.0% without dose dependence, and Bcl‐2 varied minimally (–0.4% to +3.3%), producing up to a 4.3% reduction in the Bax/Bcl‐2 ratio (Figure 4G,H). Caspase‐3 decreased slightly (1.1%–1.7%), while caspase‐9 decreased more markedly (7.3%–25.5%), indicating general suppression of apoptotic signaling (Figure 4I,J). Although AMPK‐*α* expression rose by 5.8%–19.4%, this likely reflected physiological metabolic adaptation rather than apoptosis, since proapoptotic transcription was not observed (Figure 4K). VEGF expression increased by 8.5%–33.5%, suggesting promotion of angiogenesis and preservation of function in normal cells (Figure 4L).

Overall, these findings show that PGE selectively induces apoptosis in MDA‐MB‐231 cells via AMPK‐*α*‐mediated metabolic stress, modulation of Bax/Bcl‐2, caspase activation, and VEGF suppression [28]. By contrast, in HMEC, apoptotic signaling was minimal or downregulated, highlighting PGE as a natural, selective anticancer agent. These results support its therapeutic potential and provide mechanistic insight for developing exosome‐based anticancer strategies.

### 3.5. Evaluation of Protein Expression

To confirm the reliability of the mRNA expression results and assess whether transcriptional changes were reflected at the protein level, we examined whether PGE consistently influenced apoptosis and proliferation. Cells were treated with PGE (0.0–3.8 × 10^9^ particles/mL) under the same experimental conditions, and expression of apoptosis‐ and metabolism‐related proteins was analyzed.

As shown in Figure 5A–F, in MDA‐MB‐231 cells, Bax expression rose by 30.3%–64.1% in a concentration‐dependent manner compared with N.T., while Bcl‐2 expression declined by 6.6%–55.3%, producing a 1.4‐ to 3.7‐fold increase in the Bax/Bcl‐2 ratio (Figure 5A,B). These changes support activation of the intrinsic mitochondrial apoptotic pathway and parallel the gene expression data. Treatment with 3.8 × 10^9^ particles/mL PGE also elevated the executioner caspases, caspase‐3 and caspase‐9, by 36.3% and 34.6% (Figure 5C,D), indicating that transcriptional regulation converged on downstream effector activation. Expression of AMPK‐*α*, a metabolic stress sensor interacting with the caspase pathway, increased by 24.0%–93.2% (Figure 5E), suggesting that PGE disrupted cancer cell energy homeostasis and induced apoptosis through metabolic stress signaling. In addition, VEGF, an angiogenic mediator supporting tumor proliferation, was suppressed by 8.2%–17.4% (Figure 5F), showing that PGE not only induced apoptosis but also inhibited angiogenesis, thereby restricting tumor progression through multiple mechanisms.

**Figure 5:**
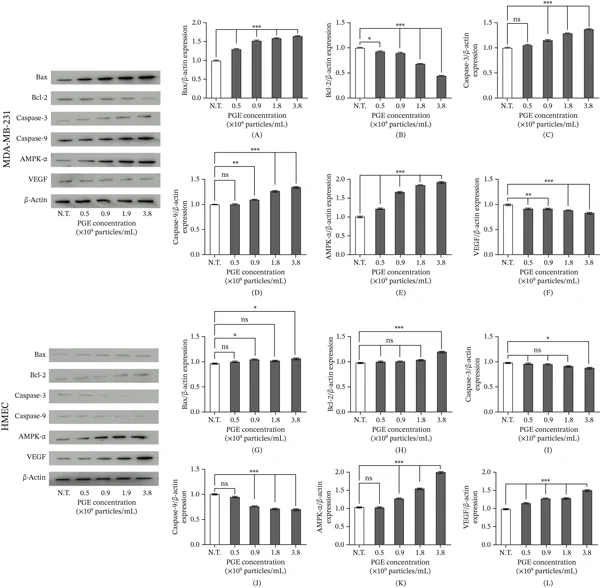
Evaluation of apoptosis‐related protein expression in (A–F) MDA‐MB‐231 and (G–L) HMEC following treatment with *Panax ginseng*–derived exosome (PGE). Expression levels of (A, G) Bax, (B, H) Bcl‐2, (C, I) caspase‐3, (D, J) caspase‐9, (E, K) AMPK‐*α*, and (F, L) VEGF were analyzed to assess mitochondrial apoptosis activation.

In contrast, HMEC showed only modest Bax variation within 12.1%, whereas Bcl‐2 increased by 20.9% (Figure 5G,H). This caused a 45.1% reduction in the Bax/Bcl‐2 ratio, which is consistent with suppressed apoptosis or maintenance of survival. Caspase‐3 and caspase‐9 expression decreased by 15.9% and 28.1% (Figure 5I,J), confirming the absence of apoptotic activation. Although AMPK‐*α* expression rose by 92.8% (Figure 5K), this is better interpreted as a physiological adjustment to preserve homeostasis rather than a proapoptotic signal [29]. VEGF expression increased by 49.9% (Figure 5L), suggesting support of vascular integrity and tissue stability. Collectively, these results show that PGE‐induced apoptosis in breast cancer cells through multiple mechanisms, raising the Bax/Bcl‐2 ratio, activating caspase cascades, stimulating AMPK‐*α* signaling, and suppressing VEGF expression, while in normal cells, these protein‐level responses were absent or opposite. These findings highlight the tumor‐specific nature of PGE action and provide a strong mechanistic foundation for its development as a selective anticancer modulator [30].

### 3.6. Evaluation of Apoptosis

Apoptosis is a fundamental biological mechanism that suppresses cancer cell growth and proliferation, and its progression can be monitored by flow cytometry using Annexin V‐FITC/PI staining [31]. In general, Annexin V binds phosphatidylserine (PS), which becomes externalized on the cell surface following loss of phospholipid asymmetry, thereby marking early apoptosis [32]. In contrast, PI penetrates cells with compromised membranes and intercalates into nuclear DNA, identifying late apoptosis or necrosis [33]. In this study, Annexin V‐FITC/PI staining followed by fluorescence‐activated cell sorting (FACS) was used to quantify whether PGE selectively induced apoptosis in MDA‐MB‐231 cells compared with HMEC.

Flow cytometric results showed that treatment of MDA‐MB‐231 cells with PGE (0.9–3.8 × 10^10^ particles/mL) reduced viable cells to 77.31%, 67.96%, and 63.17%, corresponding to a 16.77%–30.91% decline compared with the N.T. group (94.08%) (Figure 6A). Early apoptotic cells rose to 13.87%, 19.68%, and 23.61% in treated groups, versus 0.74% in N.T., representing a 13.13%–22.87% increase (*p* < 0.001). Late apoptotic populations increased to 3.85%, 8.60%, and 9.27% following treatment, compared with 0.07% in N.T., a dose‐dependent rise of 3.78%–9.20% (*p* < 0.001). As a result, total apoptotic cells accounted for 17.72%, 28.28%, and 32.88% in the treated groups, compared with 0.81% in N.T., representing a 17.91%–32.07% increase (Figure 6C, *p* < 0.001).

**Figure 6:**
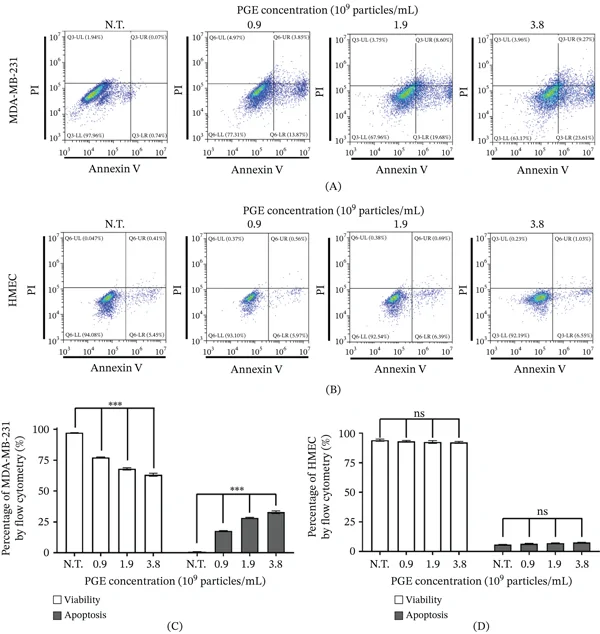
Quadrant plots illustrating Annexin V‐FITC/PI staining in (A) MDA‐MB‐231 and (B) HMEC treatment with *Panax ginseng*–derived exosomes (PGEs). Graphs showing the proportions of total apoptotic cells and viable cells in (C) MDA‐MB‐231 and (D) HMEC.

In contrast, HMEC exposed to identical concentrations showed no significant alterations. Viability was maintained at 93.10%, 92.54%, and 92.19% in treated groups, representing only a 0.98%–1.89% decrease relative to N.T. (94.08%) (*p* > 0.05). Early apoptosis was 5.97%, 6.39%, and 6.55% in treated cells versus 5.45% in N.T., a modest increase of 0.52%–1.10% (*p* > 0.05). Late apoptosis remained at 0.56%, 0.69%, and 1.03%, compared with 0.41% in N.T., corresponding to a difference of 0.15%–0.62% (*p* > 0.05). Total apoptosis in HMEC was 6.53%, 7.08%, and 7.58% after treatment, compared with 5.86% in N.T., reflecting only 0.67%–1.72% change (*p* > 0.05). Taken together, these findings confirm that PGE caused no significant cytotoxicity in normal cells, indicating minimal toxicity alongside selective apoptosis induction in cancer cells (Figure 6B,D).

Collectively, these results indicate that PGE exerts anticancer effects by selectively triggering mitochondrial‐dependent apoptosis in MDA‐MB‐231 cells via activation of Bax/Bcl‐2 and AMPK‐*α* pathways [34]. In contrast, apoptosis was not promoted in normal cells, underscoring reduced toxicity compared with conventional chemotherapeutics while retaining selective anticancer activity. To further clarify the specific mechanisms underlying PGE efficacy, additional studies on mitochondrial dysfunction, inflammatory regulation, and autophagy activation are warranted. Overall, these results confirm that PGE promotes apoptosis via Bax/Bcl‐2 signaling and provide strong evidence for its potential as a natural anticancer candidate capable of selectively inducing tumor‐specific apoptosis, thereby complementing or substituting existing therapies [35].

### 3.7. Immunofluorescence Analysis

This study sought to clarify how Bax/Bcl‐2 and AMPK‐*α* signaling activation lead to selective apoptosis in cancer cells and focused on characterizing structural changes on the cellular level. Immunofluorescence staining was therefore used to assess apoptosis by examining nuclear morphology, junctional proteins, and cytoskeletal rearrangements. Comparisons between MDA‐MB‐231 and HMEC were performed to evaluate both the biocompatibility of PGE and their ability to selectively induce apoptosis in breast cancer cells [36]. These structure–function analyses are aimed at providing morphological evidence supporting the signaling pathways regulated by PGE [37].

DAPI staining showed that, in MDA‐MB‐231 cells, increasing PGE concentrations caused marked chromatin condensation, a feature of early apoptosis [38]. Quantitative analysis revealed a 7.3%–48.4% reduction in fluorescence intensity relative to N.T. (Figure 7B), indicating nuclear instability and activation of apoptotic pathways. In contrast, no chromatin condensation was seen in HMEC (Figure 7C). Instead, PGE treatment increased fluorescence intensity by 4.1%–37.2%, suggesting nuclear stabilization and confirming the cancer cell–specific action of PGE (Figure 7D). ZO‐1 immunostaining, a marker of tight junctions often disrupted in invasive cancer, decreased by 4.1%–27.1% in MDA‐MB‐231 cells compared with N.T. (Figure 7B). Conversely, HMEC showed a 37.1%–87.6% increase in ZO‐1 after treatment, consistent with restoration of junctional integrity and potential reduction in invasiveness (*p* < 0.001). Similarly, F‐actin staining demonstrated reduced intensity by 5.1%–37.4% in MDA‐MB‐231, with filaments redistributed toward the periphery, suggesting altered cytoskeletal organization associated with apoptotic progression. However, in HMEC, we found that F‐actin intensity rose by 4.2%–61.0% relative to N.T., reflecting cytoskeletal stabilization and again highlighting divergent responses.

**Figure 7:**
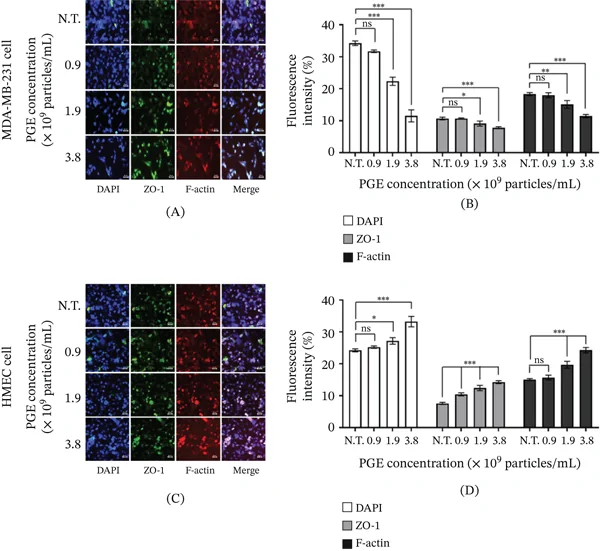
Immunofluorescence analysis of (A, B) MDA‐MB‐231 and (C, D) HMEC cells treated with *Panax ginseng*–derived exosome (PGE). (A, C) Confocal images showing nuclei (DAPI, blue), tight junction protein ZO‐1 (green), and F‐actin (red). (B, D) Quantification of fluorescence intensity for ZO‐1 and F‐actin in each cell type. Scale bars = 20 * μ*m. Fluorescence intensity was quantified using ImageJ and normalized to the number of DAPI‐positive nuclei per field after background subtraction.

Overall, these cancer‐specific structural changes align with the increased apoptosis detected by Annexin V/PI cytometry and illustrate the link between morphology and apoptotic signaling [39]. Collectively, PGE‐induced chromatin condensation, disruption of junctional integrity, and cytoskeletal reorganization in MDA‐MB‐231 cells are consistent with regulation of intrinsic apoptosis via Bax/Bcl‐2 modulation and AMPK‐*α* activation [40]. By contrast, in HMEC, PGE promoted structural stabilization, confirming both specificity for cancer cells and overall biocompatibility. These findings underscore the therapeutic potential of plant‐derived exosomes and highlight the value of integrated structural and molecular analyses in validating their efficacy.

### 3.8. Qualitative and Quantitative Analysis of Ginsenosides

To clarify the anticancer effects of PGE at the molecular level, it is necessary to identify and quantify the encapsulated bioactive constituents. Among these, ginsenosides are abundant saponin‐based phytochemicals known to mediate anticancer effects through mechanisms such as cell cycle arrest, mitochondria‐dependent apoptosis, antioxidant defense, and anti‐inflammatory activity. Accordingly, this study analyzed the composition and quantity of ginsenosides in PGE using LC‐MS/MS.

Three major ginsenosides, that is, Rf, Rh1, and Rb1, were detected in PGE. Retention times were 7.77 min for Rf, 9.92 min for Rh1, and 10.00 min for Rb1, closely matching reference standards (Figure 8). Quantitative analysis showed Rf at 2.57 mg/kg, Rh1 at 6.39 mg/kg, and Rb1 at 4.03 mg/kg (Table 2). Taken together, these findings indicate that PGE acts as a composite bioactive vesicle rather than a single‐compound entity, with ginsenosides contributing to apoptosis regulation. Notably, ginsenoside Rb1 has been reported to modulate MAPK signaling by suppressing ERK and JNK phosphorylation and to regulate the Bax/Bcl‐2 ratio, thereby promoting apoptosis [41]. The presence of Rb1 in PGE is therefore consistent with the protein expression profiles observed in MDA‐MB‐231 cells. Specifically, Rh1 is known to induce intrinsic apoptosis by increasing the Bax/Bcl‐2 ratio, activating caspases, and modulating AMPK‐*α*, all in line with the gene and protein changes seen after PGE treatment [42]. Moreover, the ginsenosides within PGE may contribute to AMPK‐*α* activation, Bax‐mediated mitochondrial permeability, and Cyt c release, and in doing so promoting apoptosome formation and caspase activation to selectively induce apoptosis in cancer cells [43].

**Figure 8:**
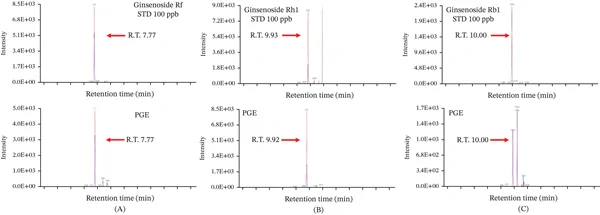
Quantitative and qualitative analysis of main compounds in *Panax ginseng*–derived exosome (PGE) based on retention time and mass‐to‐charge (*m*/*z*) using LC‐MS/MS. Relative abundance is represented as ion signal intensity in relation to total ion intensity within spectrum. (A) Ginsenoside Rf, (B) Rh1, and (C) Rb1.

**Table 2:** Retention time and multiple reaction monitoring results for identification of ginsenoside in *Panax ginseng*–derived exosome.

Compound name	Retention time (min)	Formula	Moleculur weight	Fragment (*m*/*z*)	Concentration (mg/kg)
Ginsenoside Rf	7.77	C_42_H_72_O_14_	801.02	639.53, 477.18	2.57
Ginsenoside Rh1	9.92	C_36_H_62_O_9_	638.88	477.28, 459.12	6.39
Ginsenoside Rb1	10.00	C_54_H_92_O_23_	1109.31	947.07, 459.32	4.03

This study primarily assessed PGE activity itself, without direct comparison to purified ginsenosides. Future work should include systematic evaluation of PGE versus individual constituents such as Rf, Rh1, and Rb1, both alone and in combination, to clarify their relative contributions. Such studies will improve understanding of PGE pharmacology and strengthen its translational potential as a natural, targeted anticancer therapy.

## 4. Conclusion

This study evaluated whether PGEs exert anticancer effects in breast cancer cells through apoptosis and clarified their role in selectively suppressing tumor growth. Exosome isolation yielded vesicles with an average diameter of 163.4 nm at a concentration of 1.2 × 10^10^ particles/mL, and their exosomal identity was confirmed by elevated expression of CD9 and CD63. Functional analyses showed that PGE suppressed MDA‐MB‐231 activity while preserving HMEC viability, confirming selective anticancer potential. PGE also reduced cancer cell motility while enhancing migration of normal cells, suggesting inhibition of invasion and metastasis alongside preservation of normal cell activity.

On the molecular level, PGE activated intrinsic apoptosis in breast cancer cells by elevating the Bax/Bcl‐2 ratio, increasing caspase‐3 and caspase‐9, enhancing AMPK‐*α* expression, and suppressing VEGF. Protein‐level changes mirrored gene expression, and caspase cascade activation provided direct evidence of execution‐phase apoptosis. Consistently, Annexin V‐FITC/PI staining and flow cytometry confirmed significant increases in early and late apoptosis in MDA‐MB‐231 cells, whereas HMEC showed negligible changes, reinforcing biocompatibility. Immunofluorescence demonstrated PGE‐induced chromatin condensation, reduced ZO‐1, and F‐actin reorganization in cancer cells, further supporting apoptosis induction through structural alterations.

LC‐MS/MS profiling identified ginsenosides Rf, Rh1, and Rb1 as major bioactive constituents of PGE, implicating them in the observed anticancer effects. Collectively, these findings indicate that PGE exerts potent anticancer activity by regulating AMPK‐*α* and MAPK signaling, thereby suppressing growth and motility while promoting apoptosis. These results underscore the translational promise of PGE as a functional exosome‐based anticancer material. The presence of ginsenosides, particularly Rb1, highlights their role as key mediators of efficacy and provides a strong scientific basis for developing PGEs as natural anticancer therapeutics.

## Author Contributions

Min Ho Han: conceptualization, data analysis, interpretation, and manuscript drafting. Youn Seon Hwang: visualization, validation, and data processing. Jong Hyun Oh: technical assistance and data acquisition. Do Hyeon Jin: sample preparation, resource management, and quality assurance. Ju Hwan Lee: data validation and literature curation. Jin Woo Kim: supervision, funding acquisition, and manuscript review (corresponding author).

## Funding

No funding was received for this study.

## Ethics Statement

Neither ethical approval nor informed consent was required for this study.

## Conflicts of Interest

The authors declare no conflicts of interest.

## Data Availability

The datasets generated during and/or analyzed during the current study are available from the corresponding author on reasonable request.
